# Digital Technologies-Enabled Smart Manufacturing and Industry 4.0 in the Post-COVID-19 Era: Lessons Learnt from a Pandemic

**DOI:** 10.3390/ijerph17134785

**Published:** 2020-07-03

**Authors:** Nicola Luigi Bragazzi

**Affiliations:** 1 Laboratory for Industrial and Applied Mathematics (LIAM), York University, Toronto, ON M3J 1P3, Canada; bragazzi@yorku.ca; 2 Postgraduate School of Public Health, Department of Health Sciences (DISSAL), University of Genoa, 16132 Genoa, Italy

The “Severe Acute Respiratory Syndrome Coronavirus Type 2” (SARS-CoV-2) has been identified as the infectious agent responsible for the generally mild but sometimes life-threatening communicable disease known as “Coronavirus Disease 2019” (COVID-19). Since late December, the virus has quickly spread out from its first epicenter, the city of Wuhan, province of Hubei, mainland China, where an initial outbreak had occurred, into neighboring countries and territories, becoming a global pandemic and with high death rates.

COVID-19 has significantly and dramatically overwhelmed and strained the capability of the health systems globally. Healthcare facilities and related settings have faced an unprecedented shortage of resources, both human and organizational-logistic, such as Personal Protective Equipment (PPE) and other manufactured devices, like gloves, face masks, goggles and shields, oxygen valves for respiratory machines and other ventilator components.

While COVID-19 has unearthed the limitations that plague traditional manufacturing cycles and processes, including supply chains, Manero and collaborators [1] have explored the feasibility of exploiting additive manufacturing, like three-dimensional (3D) printing, to overcome such issues. Utilizing various real-world examples, the authors have illustrated the hypes and hopes of 3D printing and how it can be deployed for counteracting and mitigating the burden generated by COVID-19.

Similarly, Salmi and coworkers [2] have concluded that 3D printing represents a “promising open source solution, especially during emergency situations”.

3D printing represents, indeed, one of the pillars of the so-called “Fourth Industrial Revolution”, together with smart sensors, nano(bio)technologies, quantum, immersive, cloud and ubiquitous computing, the “Internet of Things” (IoT)/the “Industrial Internet of Things” (IIoT), blockchain technology, next-generation telecommunication networks, Big Data Analytics and Artificial Intelligence-based approaches and algorithms, such as deep learning [3,4,5,6,7,8,9].

All these tools are highly inter-related: they enable (i) a quick and reliable detection of the spread of the virus and a containment strategy by means of contact tracing, as such enhancing the public health surveillance at the molecular and micro-level. Furthermore, they allow (ii) a personalized/individualized risk assessment, modeling and stratification of COVID-19 ascertained or suspected cases, (iii) the identification of potential strategies (behavioral, non-pharmacological, like social/physical distancing, self-isolation, quarantine, lock-down, and pharmacological, such as candidate drugs and vaccines) to be adopted and implemented by means of real-time monitoring and data collection, and, finally, (iv) the evaluation of the effectiveness of such measures.

However, the “Fourth Industrial Revolution” or “Industry 4.0” is still at its infancy and several challenges need to be properly addressed, including the high initial costs necessary for its implementation. Other issues are technical, such as the high variability in the produced material, with the subsequent need of performing different checks and trials to evaluate whether the product is properly functioning and working. Further shortcomings are given by regulatory aspects, like product certifications, which are quite challenging in the absence of an effective standardization of procedures. Moreover, even though 3D printing is an incredibly democratic technology, allowing anybody having a 3D printer to be part of big societal participatory projects (the so-called “citizen supply chain” project) [10], “unified production coalitions” and “collaborative design networks” [1], high quality of the product should be ensured. As such, there is an urgent need to establish stringent clinical safety criteria and high quality standards and protocols.

If all these issues are properly acknowledged and met, the pandemic can become the beginning of a new era and of a new society, more digitalized and more resilient to natural disasters and hazards.
